# Designing and validating a health promotion program for women with endometriosis: a mixed-methods study protocol

**DOI:** 10.3389/frph.2025.1502323

**Published:** 2025-08-26

**Authors:** Sanaz Mollazadeh, Talat Khadivzadeh, Khadijeh Mirzaii Najmabadi, Mojgan Mirghafourvand, Maryam Moradi, Javad Moghri, Leili Hafizi

**Affiliations:** ^1^ Department of Midwifery, School of Nursing and Midwifery, Mashhad University of Medical Sciences, Mashhad, Iran; ^2^ Nursing and Midwifery Care Research Center, Mashhad University of Medical Sciences, Mashhad, Iran; ^3^ Social Determinants of Health Research Center, Tabriz University of Medical Sciences, Tabriz, Iran; ^4^ Global and Women's Health, School of Public Health and Preventive Medicine, Faculty of Medicine, Nursing and Health Science, Monash University, Melbourne, VIC, Australia; ^5^ Department of Management Sciences and Health Economics, School of Health, Management and Social Determinants of Health Research Center, Mashhad University of Medical Sciences, Mashhad, Iran; ^6^ Department of Obstetrics and Gynecology, Mashhad University of Medical Sciences, Mashhad, Iran

**Keywords:** health-promoting lifestyle, endometriosis, impact of endometriosis, lifestyle, health promotion

## Abstract

**Background:**

Endometriosis is a benign and chronic gynecological estrogen-dependent disease. Research has shown that endometriosis can affect various dimensions of women's lives. It is recommended that programs be developed to improve the quality of life and promote the health of affected women. However, no existing studies have written programs to achieve these goals. Therefore, the present study aims to “design and validate a health promotion program using a logical model”.

**Methods/design:**

A mixed-methods explanatory design will be used to conduct this study in three phases. The first phase (quantitative phase) is a descriptive-analytical cross-sectional study on 200 reproductive-age women with an endometriosis diagnosis. The Health-Promoting Lifestyle Profile (HPLP-II) and Endometriosis Impact Questionnaire (EIQ) will be used to collect quantitative data. The second phase (qualitative phase) will explain women’s perceptions and experiences of the health-promoting lifestyle. In this phase, the conventional content analysis approach will be used to analyze the data. In the third phase (design of the health promotion program), findings of the quantitative and qualitative phases, the literature review, and focus group discussion (FGD) with a panel of experts will be used to develop a health promotion program based on a logical model, and its validation will be conducted using the Delphi method.

**Discussion:**

This is the first study to use a mixed-methods approach for designing a health promotion program for women with endometriosis. This study can reveal hidden issues in the attitude of patients, medical staff, and those involved in providing health services and provide a better understanding of the factors related to improving the health and quality of life of affected women. In addition, the results of research can be effective in formulating a suitable strategy that can be used by policymakers, planners, and health staff as well as respond to the needs of affected women.

## Background

Endometriosis is a common disorder in women, whose prevalence is reported between 2% and 10% in women of reproductive age. It is defined as the presence of ectopic endometrial tissue, which causes a chronic, inflammatory reaction. The most common sites of endometriotic lesion implantation in the pelvic cavity include the ovaries, uterosacral ligaments, pouch of Douglas, cervix, sigmoid colon, and pelvic peritoneum (1–3). Endometriosis may remain asymptomatic in certain patients and be accidentally discovered (4, 5). Women affected by endometriosis often suffer from associated symptoms, including infertility, periodic and non-periodic abdominal pain, painful menstruation, bloating, diarrhea or constipation, painful intercourse, painful urination, and painful defecation (6, 7). There is no definitive cure for endometriosis, and women who suffer from this chronic, estrogen-dependent disease may experience a range of symptoms, from mild pain to very debilitating disease. The etiology of endometriosis is intricate and multifactorial (8, 9). Although the exact cause of endometriosis is still unclear, retrograde menstruation is widely acknowledged as a significant contributing factor (10, 11). The results of qualitative studies that have investigated the impact of endometriosis on women's lives have shown extensive effects in different dimensions, such as physical health, psychological well-being, social interactions, sexual and marital relationships, economic factors, employment and occupational dimensions, education, and lifestyle (1, 12, 13). Health promotion is defined as “the procedure of enabling individuals to increase control over and to improve their health” (14). Health-promoting lifestyles are among the factors of health that are known as fundamental factors in disease prevention. Having a healthy lifestyle and carrying out a health-promoting lifestyle should be considered as the main strategies for improving and maintaining health (15). Considering that endometriosis is a chronic disease that has no definitive treatment and significantly affects women's lives, it is necessary to consider health-promoting lifestyles and concerns of endometriosis to get widespread information that can improve the well-being of affected women. Unfortunately, endometriosis is a profoundly distressing experience, and women's problems are underdiagnosed, so the lasting consequences of the situation are ignored and lead to long-term distress (16). Therefore, adopting a healthy lifestyle seems as a useful and essential approach for women with endometriosis (4, 6, 17).

## Study aim

The results of this study will provide better understanding of women's understanding and experience of endometriosis and health-promoting lifestyle and a deep image, detailed information according to their culture and expectations, resulting in designing a health promotion program to improve the quality of life of women affected by endometriosis.

### Specific objectives

#### Main objective

To design and validate a health promotion program for women with endometriosis.

#### Main research question

What are the dimensions of a health promotion program in women with endometriosis?

### General objectives of study phases (quantitative, qualitative, and program design)

#### First phase (quantitative)

Determining a health-promoting lifestyle and its relationship with the effects of the disease in women with endometriosis.

#### Second phase (qualitative)

Exploring the perception and experience of women with endometriosis from a health-promoting lifestyle.

#### Third phase (design and validation of the program)

Designing and validating the health promotion program for women with endometriosis.

### Quantitative phase-specific objectives

1. To determine the status of health-promoting lifestyle and its subdomains (health responsibility, physical activity, nutrition, spiritual growth, interpersonal relationships, and stress management) in women with endometriosis who are referred to the endometriosis clinic
2. To determine the effects of endometriosis on the lives of women with endometriosis who are referred to the endometriosis clinic
3. To determine the relationship between a health-promoting lifestyle and the effects of endometriosis in women.

### Specific goals of the program design phase

1. To design a health promotion program for women with endometriosis
2. To validate of health promotion program in women with endometriosis

## Methods/design

### Study design

A mixed-methods sequential explanatory design will be used to conduct this study by collecting, analyzing, and integrating the quantitative and qualitative data. Mixed methods are based on the principles of the pragmatism paradigm, based on which, the integration of quantitative and qualitative approaches enhances our understanding of an issue or problem. In this study, quantitative and qualitative data will be collected in the first and second phases, respectively. The qualitative data will be expanded, and the collected quantitative data in the first phase and both quantitative and qualitative data will be integrated into the Discussion section and will be used to develop the health promotion program (18, 19).

### Phase one: quantitative study

A descriptive-analytical cross-sectional study will be carried out. Women at reproductive age (15–49 years) diagnosed with endometriosis, who were seeking care at the endometriosis clinic of Imam Reza Hospital in Mashhad, Iran, will be recruited. The Obstetric and Reproductive Questionnaire, Health-Promoting Lifestyle Questionnaire (HPLP-II), and the Endometriosis Impact Questionnaire (EIQ) will be used to collect quantitative data. The relationship between health-promoting lifestyle and its subdomains and the impacts of endometriosis on various aspects of women's lives will be determined.

#### Sample size and sampling method

The sample size was determined based on both specific objectives related to a health-promoting lifestyle and the impact of endometriosis. Based on the results of Abd-Elaziz Ibrahim et al.'s study (6) and taking into account the largest standard deviation (SD = 3.74) observed in the health-promoting lifestyle subdomains, typically in the spiritual growth subdomain, with *α* = 0.05, power = 90%, and *d* = 0.05, a sample size of 186 women calculated around a mean of 15.32 was obtained. Moreover, based on the results of Moradi et al.'s study (20) and taking into consideration the largest standard deviation of disease impacts subdomains (SD = 32.7) for the fertility subdomain, with *α* = 0.05, power = 90%, and *d* = 0.05, a sample size of 196 women calculated around a mean of 65.28 was obtained. Given that the sample size determined based on the variable of disease impact was larger, the final sample size was calculated to be 196 women, rounded to 200.

#### Sampling

The present study was confirmed by the Ethics Council of Mashhad University of Medical Sciences (ethics code: IR.MUMS.NURSE.REC.1401.064). Subsequently, data collection will commence at the endometriosis clinic of Imam Reza Hospital in Mashhad. The researchers will attend the endometriosis clinic, and eligible women will be identified by referring to their medical records and invited to participate in the study using convenience sampling based on the inclusion and exclusion criteria. Before the study, written informed consent will be obtained from the participants. The researchers will introduce themselves to the patients and provide them with necessary explanations regarding the study's purposes. Then, the patients will be given written questionnaires to complete.

#### Data collection tools

Data will be collected using the Health-Promoting Lifestyle Profile II (HPLP-II) (21) and Endometriosis Impact Questionnaire (EIQ) (20). In addition, the researchers will develop sociodemographic and obstetric questionnaires, including inquiries on age, education, occupation, income sufficiency for expenses, first-degree relatives, obstetric and reproductive information, and history of smoking/hookah use, alcohol consumption, chronic diseases, autoimmune diseases, and allergies.

#### Inclusion criteria

Study participants will be women with a confirmed endometriosis diagnosis and treatment records from an endometriosis clinic (women aged 15–49 years diagnosed with endometriosis by open surgery, laparoscopy, histological examination, presence of endometrioma cyst, or diagnosis via ultrasound and MRI confirmed by a gynecologist). Moreover, women will be involved in the study if they met the following: diagnosed with endometriosis localized to the pelvis and peritoneum, experienced the onset of endometriosis symptoms at least 1 year, diagnosed with no presence of endometriosis in the partial region or remote organs, (e.g., lungs and brain), had Iranian nationality, married, literate to answer questions, non-menopausal status (amenorrhea for over a year), and did not suffer from any other major diseases, such as mental disorders, severe depression, schizophrenia, or chronic diseases, such as diabetes, kidney disease, rheumatological disorders, cancer, and life-threatening diseases.

Contrary to the results of some studies suggesting no relationship between the severity or stage of endometriosis and the severity of the symptoms (22–25), we will not consider the stage of the disease as an inclusion criterion in this study.

#### Exclusion criteria

The women who decline to further cooperate and submit an incomplete questionnaire (with >10% of the questions left unanswered) will be excluded from our study.

#### Scales and data collection

The HPLP-II questionnaire, as a standard questionnaire for examining health-promoting lifestyles, includes 52 questions in six dimensions: nutrition, physical activity, spiritual growth, health responsibility, stress management, and interpersonal relationships. All items are scored on a Likert scale ranging from 1 to 4 (1 = never, 2 = sometimes, 3 = often, 4 = always) (26). This questionnaire has been used in numerous previous studies (6, 17, 27). The English version of the HPLP-II questionnaire was translated into Persian, and its validity and reliability were assessed in earlier studies in Iran (24, 28). The Cronbach's alpha for the questionnaire was reported as 0.87 in a study on the elderly population aged 65 years or older ((29).

Another instrument used in this study is EIQ, which was originally developed and psychometrically analyzed by Moradi et al. (20). EIQ was initially designed in Australia and applied for the first time in Iran. This questionnaire aims to measure the long-term impacts of chronic endometriosis on various aspects of women's lives with endometriosis. Comprising 63 items, it assesses the impact of the disease on multiple dimensions of the affected women's lives, including the impact of the disease on physical, psychological, social, marital intimacy and sexual relationships, fertility, occupational and financial aspects, education, and lifestyle across three distinct periods (the last 12 months, 1–5 years ago, and >5 years ago). The English version of the EIQ questionnaire was translated into Persian, and its validity and reliability were assessed in earlier studies in Iran. CVI and CVR values of the EIQ tool were 0.97 and 0.94, respectively. The item-to-total correlation confirmed the construct validity of all seven dimensions of the tool, more than the cutoff (0.3), except for lifestyle. Cronbach's alpha coefficient and intracorrelation coefficient (ICC) were acceptable for all dimensions (9).

While other instruments, such as the EHP-30, assess quality of life over shorter periods (e.g., the past 4 weeks), the EIQ uniquely captures the chronic and recurrent nature of endometriosis by focusing on long-term effects. Similar to the ETSQ and EPBD, the EIQ was developed using patient input from focus groups and interviews, mirroring the process used for the ECQ, albeit with different developmental methods. Furthermore, the EIQ includes unique subscales for education and lifestyle, while the EHP-30 features subscales relating to relationships with children, medical professionals, and therapy (30).

#### Data analysis

Data will be analyzed using SPSS 24 software. The normality of quantitative variables will be investigated using the Shapiro–Wilk test. Descriptive statistics, including frequency (percentage), mean (standard deviation) for normal data, and median (25th–75th quartile) for non-normal data, will be used to describe participants’ sociodemographic and reproductive characteristics of affected women. Bivariate tests, including Pearson's or Spearman's correlation, will be used to determine the relationship between a health-promoting lifestyle and its subdomains and the impacts of endometriosis on various aspects of women's lives. *P* < 0.05 will be considered statistically significant.

### Phase two: qualitative study

The second phase consists of an exploratory qualitative study with content analysis. This qualitative method will be used to explore and explain the understanding and experience of the health-promoting lifestyle and the effects of the disease in women with endometriosis.

#### Sampling method

Based on the results of the average score for overall health-promoting lifestyle and the effects of endometriosis obtained in the quantitative section, participants in the qualitative part consisted of extreme cases (31), that is, those who will gain 10% of the upper and lower limits of the overall health-promoting lifestyle in the quantitative part will be selected. The Consolidated Criteria for Reporting Qualitative (COREQ) checklist will be used for reporting the results of this study. A conventional qualitative content analysis approach will be adopted, which allows for obtaining direct information from a study without imposing preconceived categories or theories.

#### Data collection

To collect qualitative data, semi-structured in-depth interviews with open-ended questions will be conducted. During the interviews, the researchers must encourage participants to express their views and experiences freely (31). Prior to the qualitative stage, an interview guide will be developed by designing questions based on the findings of the quantitative phase and related factors. To obtain credible data, the researchers must begin the interviews with predesigned questions, analyze responses to each question, and raise in-depth and exploratory questions such as “What do you mean?”, “Why?”, “Please explain further,” and “Please give an example.” All interviews will be conducted in health centers chosen by the participants. Sampling continued until data saturation is reached, that is, until no new information or code is received (32).

#### Data analysis

A conventional qualitative content analysis approach will be adopted to analyze the data. In this approach, the researchers read and interpret all available texts to gain a complete understanding of them. Then, the texts are examined word-for-word to extract relevant codes. The main advantage of this approach is obtaining direct information from a study without imposing preconceived categories or theories (33). Data will be analyzed based on a qualitative content analysis method introduced by Graneheim and Lundman (34). This method allows for extracting not only the explicit content of the texts but also their implicit content with varying degrees of abstraction. We will use four criteria to evaluate the accuracy of the qualitative data (credibility, dependability, confirmability, and transferability) (35). The interview texts and codes will be organized in MAXQDA 20.

#### Integration of quantitative and qualitative data

In this study, integration will be used in the design of a health promotion program for women with endometriosis by combining findings from the qualitative and quantitative phases, as presented in the Discussion section. Some researchers who use this method present a paragraph that reports the quantitative results and then continue with a paragraph that deals with the qualitative results. Some also state a descriptive quantitative result and then immediately include a qualitative quote in the same paragraph to confirm the quantitative result (36).

### Phase three (design and validation of the program)

After conducting a quantitative and qualitative study, in the third stage of the current study, the research team intends to design and validate a health promotion program for women with endometriosis using a logical model. Designing and implementing health promotion programs and providing health-promoting lifestyle solutions can have a significant effect on improving the quality of life, increasing life expectancy, and reducing health care costs (37). The logical model has been used for planning and evaluating health promotion projects. In the field of public health and health promotion, the logical model is considered a valuable tool. Logical models show the relationship between program input and desired activities and results. Logical models are dynamic and respond to the specific needs of programs and innovations (38).

#### Developing a health promotion program

In this study, based on the results of the quantitative stage and the codes that will be explained based on the needs and challenges of the affected women in the qualitative stage, it should be determined to what extent the data obtained from the studies are aligned or inconsistent and to what extent the data obtained from the two phases of the study support each other or are in conflict with each other (39). Then the review of the literature will be done, and the extraction of needs will be done at the same time. The extracted needs are again confirmed by the participants and listed based on the participants’ prioritization. Based on the categories of needs, solutions are listed by taking into account the opinions of the participants and available experts related to this issue (reproductive health specialists, midwives, and gynecologists) through a focus group discussion to be used in the third phase by using a logical model to design a health promotion program. A logic model is a program planning tool that defines the inputs, outputs, and results of a program. Logical models are visual tools that can help programs create action plans for activities. It explains the thinking behind program design and shows how specific program activities lead to desired results. Inputs include resources, activities, and investments made in a program. Outputs include activities, services, events, products that reach the main audience of the program, and the results of changes related to the intervention of the program that are experienced by the primary audience (40).

#### Steps of developing a logical program

1. Identifying the problem, collecting data, and extracting needs
2. Stakeholder analysis
3. Definition of program elements
4. Making and drawing the model
5. Program approval and accreditation (28).

After designing the “health promotion program for women with endometriosis” based on the logical model, validation will be conducted using the “Delphi method.” Obtaining the views and opinions of experts in the field of health is very important for policymakers in a targeted and scientific process.

#### Delphi’s steps

First stage: The panel of experts will be selected to participate in the activities, the reason and logic of the study will be explained to them, and they will be requested to participate in the study.

Second stage: Each of the participants will be asked to answer to provide a list of value judgments, forecasts, or opinions about the issues or topics determined.

Third stage: The answers will be collected and integrated into a questionnaire for use in the first round of the study. Then the members of the same group are asked to rank the recorded statements in order of priority or importance.

Fourth stage: As soon as all the questionnaires of the participants of the first round are received, the data will be analyzed and analyzed statistically. Then, the statements are readjusted based on the obtained ranks, in the form of another questionnaire to be used in the second round of the study.

Fifth stage: The second round questionnaire, along with the statistical summary of the first stage, is sent to the members of the same group, and they are asked to answer the second questionnaire based on it.

Sixth stage: The fifth stage is repeated for the third round questionnaire. At the end of the third round, the examiner prepares a report of the ranked statements and determines how many changes have been made in the views. Then, the social opinions, along with other related recommendations received from the participants, are summarized and presented to the beneficiaries for decision-making.

## Discussion

This study, the first in Iran, employs a sequential explanatory mixed-methods approach to design and validate a health promotion program for women with endometriosis. This approach, offering a more comprehensive understanding than purely quantitative or qualitative methods (31), begins with a quantitative, cross-sectional analysis examining the relationship between health-promoting lifestyle scores and the impact of endometriosis on various aspects of women's lives. The subsequent qualitative phase explores women’s perceptions of a health-promoting lifestyle. These qualitative findings will elucidate and expand upon the quantitative results, identifying specific health-promoting behaviors, associated feelings and perceptions, and key influencing factors. Finally, the quantitative and qualitative findings will be integrated through focus group discussions and literature review to develop and validate the health promotion program using a logic model.

According to the Healthy Lifestyle Questionnaire, various dimensions play a role in a healthy lifestyle, which consist of nutrition, physical activity, spiritual growth, health responsibility, stress management, and interpersonal relationships. Studies aimed at investigating the relationship between the consumption of certain foods or lifestyle modifications are limited. The results of a study showed that increasing the consumption of some fruits, omega-3 fatty acids, and dairy foods may reduce the risk of endometriosis. Modification of diet and lifestyle and how they relate to the risk of endometriosis and/or symptoms related to endometriosis are discussed (38). To improve dietary patterns, appropriate changes should be made in knowledge, attitude, and performance, in addition to the environment and food consumption. Improving the nutritional status as a primary prevention helps to improve health and promote the quality of life at all ages. Nutritional therapy as secondary and tertiary prevention is an effective way to manage and control the disease, which reduces the risks of chronic disease, slows down the progression of the disease, and reduces its manifestations (23). It seems that participating in physical and occupational activities has been accompanied by changes in the synthesis of sex steroids, a decrease in the level of estrogen, and an increase in globulins that bind to sex hormones, which may increase the risk of hormone-related diseases such as endometriosis (39, 40). While review articles have not definitively established exercise and physical activity as treatments for endometriosis symptoms, their potential benefits warrant consideration, especially given the chronic nature of the disease and the recommendation for long-term clinical studies. A systematic review and meta-analysis suggest that physical activity and exercise improve quality of life, pain intensity, mental health, pelvic floor dysfunction, and bone density. Therefore, women with endometriosis should be aware of the potentially beneficial role of exercise and physical activity (41).

Human spiritual development is a health-promoting behavior. Beyond the physical, humans possess a soul or spirit that reflects their true essence. Spirituality, perfectionism, and theism are key to understanding human growth. Spirituality connects individuals to a higher power, providing purpose and meaning (42). While research is limited, spirituality and spiritual awakening may aid in managing conditions such as endometriosis in women (43, 44). Endometriosis significantly diminishes the quality of life for affected women. Misdiagnosis or delayed diagnosis, inappropriate treatments, and unnecessary surgeries by non-specialists can lead to long-term, irreparable complications. Dissatisfaction with the chronic treatment process is common. Endometriosis-related infertility, coupled with the stress of medication and potential treatment failure, can cause psychological harm. The disease negatively impacts interpersonal relationships, social participation, sexual function, and occupational performance, with physical and psychological issues further hindering educational and professional success (45).

Stress management is crucial for health-promoting behaviors, as stress—physical, mental, and emotional reactions to life's changes—becomes a growing public health concern. Women report higher stress levels than men (46), and diseases such as endometriosis can significantly impact mental health, creating a psychological burden. Research indicates a bidirectional relationship between stress and endometriosis: stress may exacerbate the condition, while endometriosis can contribute to stress and anxiety (47, 48).

Endometriosis has a significant impact on the quality of life and daily activities of affected women, including interpersonal relationships and sexual desires, as well as the ability to do daily tasks, work, and fertility, interruptions in education and work, reduced social participation, and physical and mental well-being. The main goal of health promotion is to support healthy behavior through a multidimensional approach. Healthy behavior is not only a physical activity but a combination of mental, educational, and environmental factors that enable us to live a healthy life (28–41). Unfortunately, even though life with endometriosis is very difficult for many patients, the problems of these patients have not been given much attention, and women suffer from the harmful effects of this disease for a long time (1).

Patients with endometriosis face a world full of false information about their disease, taboos, unanswered questions, a lack of timely diagnosis, and problematic treatments, which are covered with a painful, stubborn, and chronic disease (6). The results of experimental trial studies, conducted by the health promotion and health education model, showed a positive effect on improving the lifestyle of women with endometriosis and increasing information to improve the lifestyle and reduce the pain caused by endometriosis. Therefore, adopting a healthy lifestyle for women with endometriosis seems useful and necessary. These studies suggest providing information in the majority of a written program with a clear and concise explanation for these patients in future studies (4, 5, 14).

Health responsibility, a key component of a healthy lifestyle, involves actively managing one's well-being through weight control, abstaining from smoking, and adhering to medical advice (49). This proactive approach enhances overall well-being and reduces healthcare costs. For chronic conditions like endometriosis, which significantly impacts physical, psychological, and financial well-being, a single-provider model often proves insufficient. Many women with endometriosis experience persistent pain due to the lack of coordinated, multidisciplinary care involving psychology, nutrition, pain management, and physiotherapy. Therefore, exploring patient-centered, multidisciplinary care models is crucial to improving long-term outcomes for women with endometriosis across various healthcare systems. Further research is needed to evaluate the effectiveness and value of multidisciplinary care compared with the single-provider approach (50).

Interpersonal relationships significantly impact health, influencing thoughts, expectations, perceptions, and reactions (49). Positive relationships within families and communities foster shared understanding and improve psychological well-being, reducing distress and anxiety (51). However, conditions such as endometriosis can strain interpersonal and marital relationships, negatively affecting daily life, social well-being, general health, productivity, and self-esteem in affected women (52, 53).

A study found that increasing general knowledge among women with endometriosis through support-educational guides and mass media significantly reduced pain and improved their quality of life. Utilizing educational aids in healthcare settings, mass media, or community outreach appears to enhance interpersonal and social relationships, leading to improvements in all dimensions of life for women with endometriosis compared with a control group (54).

The World Health Organization states that promoting health includes encouraging a healthy lifestyle, creating supportive environments for health and strengthening community performance, changing the direction of health services, and creating optimal policies in public health (42).

This mixed-methods, sequential-descriptive study will develop and validate a health promotion program for women with endometriosis in three stages: quantitative, qualitative, and program design/validation. Endometriosis, a chronic gynecological disease, significantly impacts various aspects of women's lives, necessitating its prioritization in healthcare. Research in Iran and globally highlights the disease's detrimental effects on physical, sexual, marital, psychological, social, economic, occupational, educational, and lifestyle domains, as well as its economic burden on individuals and society. This study will address these multifaceted impacts and needs in the development and validation of the health promotion program. However, given the study's location in Mashhad, a major Iranian city, findings may not be generalizable to women with endometriosis in rural areas.
